# Tight junction disruption through activation of the PI3K/AKT pathways in the skin contributes to blister fluid formation after severe tibial plateau fracture

**DOI:** 10.3389/fbioe.2022.946261

**Published:** 2022-08-11

**Authors:** Jialiang Guo, Xiaojun Chen, Zhe Lin, Lin Jin, Zhiyong Hou, Weichong Dong, Yingze Zhang

**Affiliations:** ^1^ The School of Medicine, Nankai University, Tianjin, China; ^2^ Department of Orthopaedics, The Third Hospital of Hebei Medical University, Shijiazhuang, China; ^3^ Department of Pharmacy, The Second Hospital of Hebei Medical University, Shijiazhuang, China; ^4^ Chinese Academy of Engineering, Beijing, China; ^5^ NHC Key Laboratory of Intelligent Orthopeadic Equipment (The Third Hospital of Hebei Medical University), Shijiazhuang, China

**Keywords:** tibial plateau fracture, fracture blisters, tight junctions, acute compartment syndrome, paracellular pathway

## Abstract

**Background:** Acute compartment syndrome (ACS) is an orthopedic emergency that commonly occurs after severe tibial plateau fracture. Fracture blisters form on the skin, and it was found in our previous study that when blisters form, the compartment pressure significantly decreases. However, the potential mechanism underlying this pressure decrease has not yet been elucidated.

**Methods:** To obtain a comprehensive understanding of the changes that occur after blister formation on the skin, the changes in tight junction expression in the skin after tibial plateau fracture were observed. Blister samples and normal skin were collected from patients with bicondylar tibial plateau fractures with or without blisters. The epidermis thickness was measured, and the difference in the levels of K1, K5, K10, and skin barrier proteins such as claudin 1, claudin 2, and occludin between the two groups was evaluated by immunochemistry analysis, immunofluorescence, Western blotting, and qPCR.

**Results:** The skin was thinner and the levels of K1, K5, and K10 were significantly decreased in blistered skin. Furthermore, the PI3K/AKT pathway was found to be activated, and the tight junction expression was significantly decreased in blistered skin. This indicates that the paracellular pathway, which is essential for accelerating fluid accumulation in blisters and indirectly decreases compartment pressure, was activated.

**Conclusion:** Changes in the tight junction expression after blister formation may underlie blister fluid formation and indirectly explain the decrease in compartment pressure under blistered skin after severe tibial plateau fracture.

## Introduction

The treatment of severe tibial plateau fractures after high-energy trauma in adults is challenging. Acute compartment syndrome (ACS) is always considered an orthopedic emergency that is commonly encountered in severe tibial plateau fractures. Among the numerous physical signs after fracture, blisters on the skin have been reported to be a common indicator found in approximately 2.9% of all fractures requiring hospitalization (Uebbing et al., 2011). Two types of fracture blisters have been observed: one is a blood-filled blister, which represents more severe injury to the skin with complete separation of the dermo-epidermal junction; and the other is the serous-filled blister that exhibits only partial separation of the dermo-epidermal junction (Strebel et al., 2020).

A fracture blister is a phenomenon observed on the skin, and its importance has generally been ignored in orthopedic and dermatologic studies. The presence of these blisters frequently alters the proposed treatment or timing of surgery. The reason for these changes is the fear of wound breakdown or surgical site infection when an incision is made through a blister bed. In our previous research, it was found that when blisters appeared, the compartment pressure was lower than when they did not appear (Guo et al., 2021). It was therefore assumed that the formation of skin blisters might be a potential method of myofascial pressure release (Guo et al., 2019). However, whether the skin can release or transmit the abnormally increased tension or pressure originating from the compartment through its own regulation is still unknown. Therefore, an exploration or observation of the changes after blister formation on the skin would be beneficial to explain the reason for the decreased compartment pressure.

The skin, which comprises the epidermis, dermis, and hypodermis, can protect our body from harmful or poisonous external environments. The epidermis is the outermost layer composed of multilayered epithelial tissue, and the barrier function of the skin is mainly provided by the corneocytes that are connected through corneodesmosomes and lamellar lipids in the stratum corneum (Jung et al., 2019). Furthermore, tight, gap, and adherens junctions also contribute to the protective skin barrier (Draelos, 2012). Among them, tight junctions (TJs) create an intercellular barrier limiting the paracellular movement of solutes and material across epithelia. In general, the architecture can be conceptualized into compartments with transmembrane barrier proteins (claudins, occludin, etc.), linked to peripheral scaffolding proteins. Furthermore, there has been gradual recognition among physiologists studying epithelial transport that TJs, which have been considered to be impermeable structures, are actually variably permeable to ions and solutes (Frömter and Diamond, 1972). Many studies have focused on TJs and their regulators as therapeutic targets (Gamero-Estevez et al., 2019; Lobo de Sa et al., 2019; He et al., 2020). Quercetin, a common flavonoid was reported to improve the intestinal TJ barrier and reduce the rate of kidney stone formation by inducing phosphatidylinositol-3-kinase (PI3K)/protein kinase B (AKT)-regulated claudin expression changes (Gamero-Estevez et al., 2019).

It was previously reported that there are three distinguishable processes when blisters occur: the loosening of the structure, a phase of discontinuity, and fluid accumulation (Bork, 1978). However, no experiments on TJ changes in blistered skin in tibial plateau fractures have been conducted, and the pathways for fluid migration or accumulation remain unknown. To obtain a comprehensive understanding of the TJ changes in blistered skin in tibial plateau fractures, this research collected the patient skin from bicondylar tibial plateau fractures and explored the TJ changes in the blistered skin with the objective of explaining the reasons for the potential ability to release or transmit the abnormally increased tension or pressure originating from the fractured compartment.

## Materials and methods

### Demographic data

**FIGURE 1:**
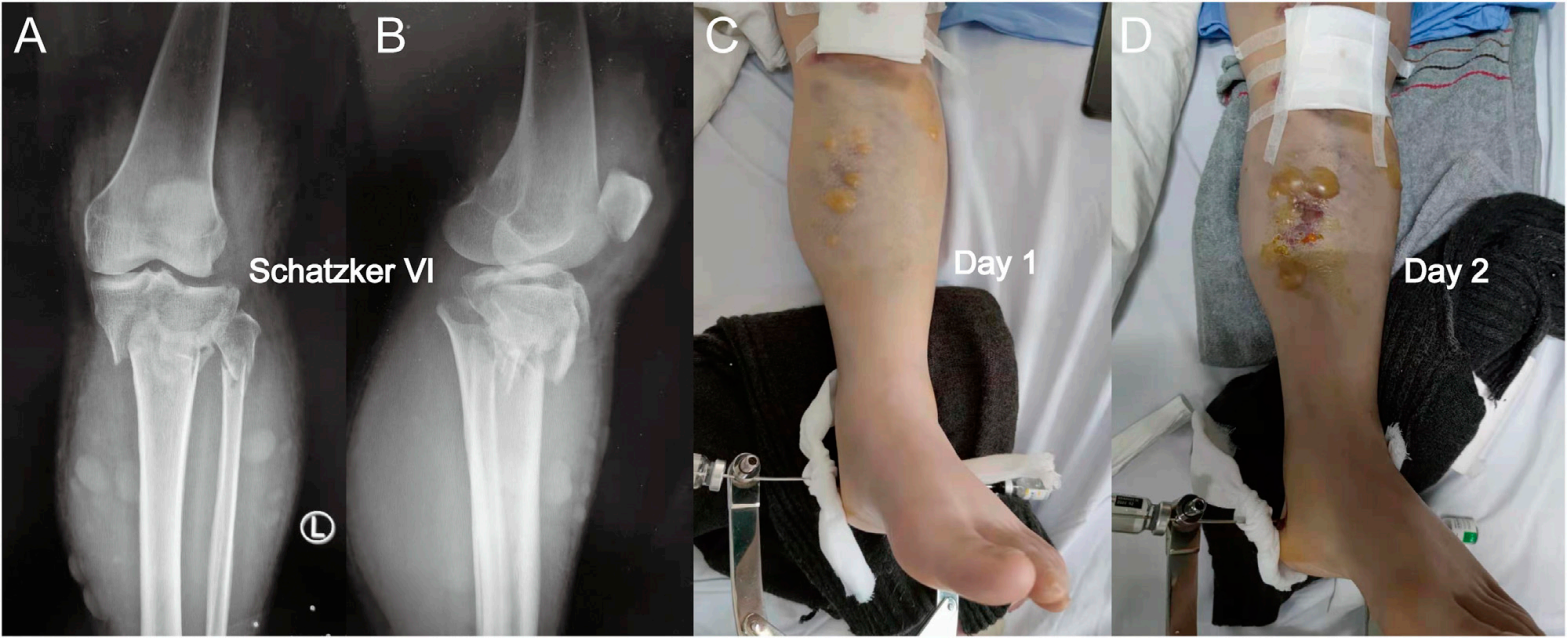
**(A)** Anterior-posterior X-rays of a Schatzker VI tibial plateau fracture. **(B)** Lateral view of this fracture. **(C)** Fracture appearance with a serous-filled blister observed on the skin around the fracture site (proximal tibia) after admission. **(D)** Blister became larger after 1 day, and the patients were not subjected to fasciotomy. When a blister was observed, the intracompartmental pressure decreased significantly.

**FIGURE 2:**
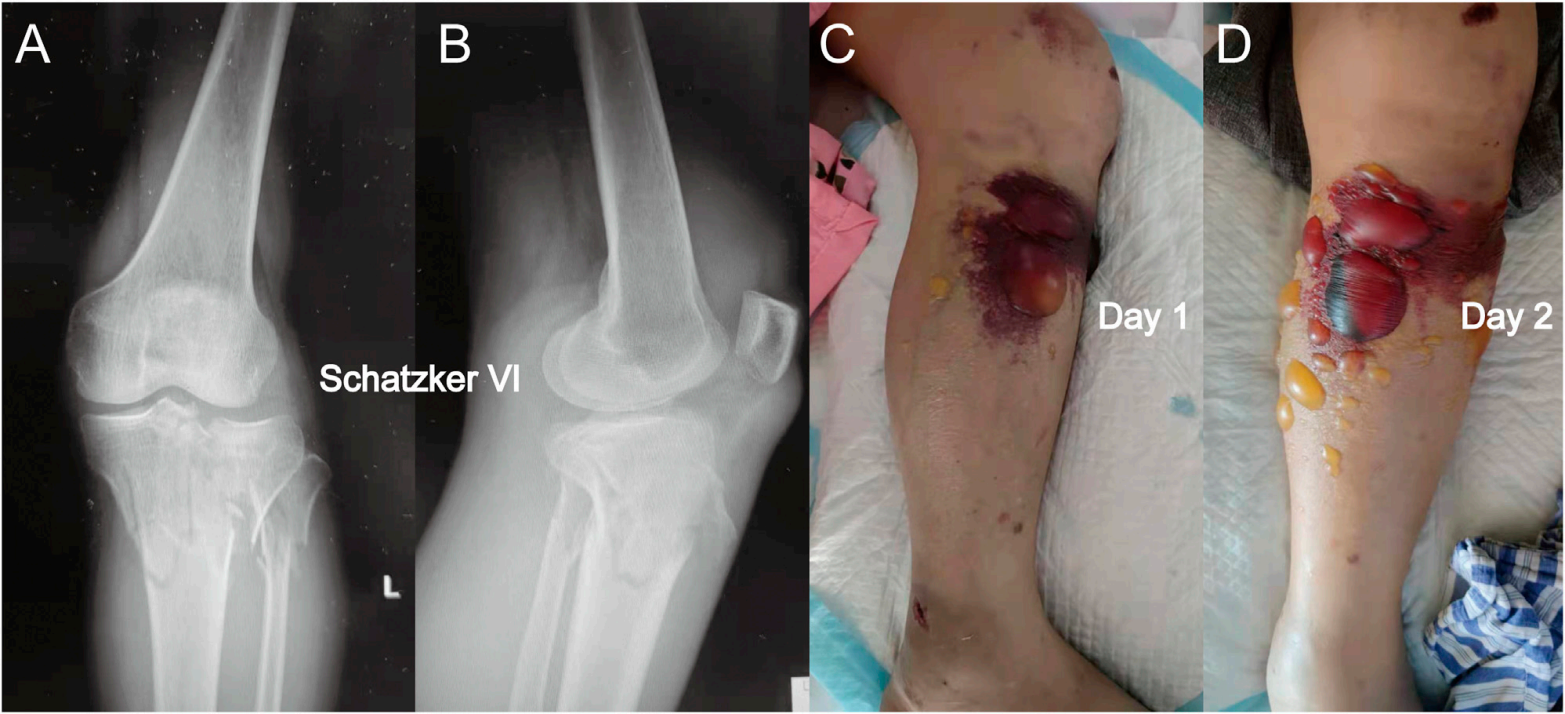
**(A)** Anterior-posterior X-rays of another Schatzker VI tibial plateau fracture. **(B)** Lateral view of this fracture. **(C)** Fracture appearance with a blood-filled blister. The blood-filled blister was larger than the serous blister observed in Figure 1. **(D)** Blister became larger after 1 day, and a similar phenomenon in which the intracompartmental pressure decreased after the appearance of the blood-filled blister was observed.

The medical records for severe tibial plateau fractures with or without ACS (Schatzker V and VI) in our department were included in this research. This study was approved by the Regional Ethics Committee of the Third Hospital of Hebei Medical University (S2020-022-1) and was performed in accordance with the ethical standards in the 1964 Declaration of Helsinki. The clinical trial number of this research was NCT04529330. The inclusion criteria were as follows: fracture patients with Schatzker V or VI who were older than 18 years. The exclusion criteria were pathologic or extra-articular proximal tibial fracture and those patients with 18 years of age or younger. Normal skin (10 samples from individuals with tibial plateau fractures who did not have blisters, control group, CG) and blistered skin (12 samples from individuals with tibial plateau fractures who had blisters, blister group, BG) were collected from the operation procedure (Figures 1, 2), and informed written consent was obtained from all of the enrolled patients. There were no significant differences in demographic data between the two groups (Table 1). The blister and normal tissues were collected around the main fracture line position, and the size of each sample was approximately 1 cm^2^. Each tissue sample was divided for use in various assays.

**TABLE 1:** The demographic data in CG and BG.

	CG	BG	*P*
**Gender**			
Male	7	10	—
Female	3	2	0.406
**Age**	46.3 ± 7.6	45.9 ± 7.0	0.821
**Fracture Type**			
Schatzker V	4	7	—
Schatzker VI	6	5	0.392
**Chronic Disease**			
Normal	5	7	—
Hypertension	3	4	—
Diabetes	2	1	0.729
**Acute complications**			
Artery injury	0	0	—
VTE	3	1	0.293
Open injury	0	0	—

### Histopathology

A 4% paraformaldehyde PBS (pH 7.4) solution was utilized to fix the skin for 1 day at 4°C. The skins were then processed for standard dehydration in graded alcohol and embedding in paraffin. Sagittal 3 μm sections were cut (Microm HM360, Waldorf, Germany), and hematoxylin and eosin (HE) staining was used for histological evaluation with standard methods. Light microscopy (Nikon eclipse C1, Nikon, Japan) was used to obtain histological images, which were processed with built-in software (Nikon DS-U3).

### Immunohistochemical staining

Normal or blister human skin samples were cut into 3 µm slices, and a rehydration procedure was sequentially applied with a descending ethanol series. Next, a high-pressure cooker was used to conduct antigen retrieval (pH 8.0, Servicebio, G1106, Wuhan, China) for 20 min, followed by three cooling phases over 5 min. To block endogenous peroxidase activity, the sections were placed in 3% hydrogen peroxide and incubated at room temperature in the dark for 25 min. The sections were placed in PBS (pH 7.4) and shaken on a decolorizing shaker 3 times for 5 min each time. Bovine serum albumin (BSA; 3%) was added to the circle to evenly cover the tissue, and the tissues were sealed for 30 min at room temperature. Anti-keratin1 (Servicebio, GB14050, 1:100, Wuhan, China), anti-keratin 5 (Servicebio, GB14016, 1:100, Wuhan, China), and anti-keratin 10 (Servicebio, GB14051, 1:100, Wuhan, China) antibodies were used to evaluate the difference between the two groups. After incubation with the primary antibodies, the sections were placed in PBS (pH 7.4), washed by shaking on a decolorizing shaker three times (5 min each time), and incubated with secondary antibody (HRP-labeled) at room temperature for 5′ minutes. Hematoxylin (Sigma, St. Louis, MO, United States) was used for nuclear staining. All immunohistochemistry images were obtained and analyzed by microscopy and with a relative imaging system (Nikon E100).

### Immunofluorescence staining

Commercial primary antibodies, including anti-occludin (Servicebio, GB-111401, 1:500, Wuhan, China), anti-claudin 1 (Servicebio, GB14066, 1:100, Wuhan, China), anti-claudin 2 (Servicebio, GB14068, 1:100, Wuhan, China), anti-keratin 1 (Servicebio, GB14050, 1:100, Wuhan, China), anti-keratin 5 (Servicebio, GB14061, 1:100, Wuhan, China), and anti-keratin 10 (Servicebio, GB14051, 1:100, Wuhan, China) were diluted. After incubation with the primary antibodies, the samples were washed with PBS three times (5 min each time). Next, the samples were incubated with secondary antibodies at room temperature for 5′ min in darkness. After three washes with PBS (5 min each time), DAPI (Sigma, St. Louis, MO, United States) was used for nuclear staining. All IF images were obtained by microscopy and with a relative imaging system (Nikon Eclipse C1). To quantify the level of protein expression, ImageJ software was utilized to generate a mask, which was applied to each corresponding protein channel being quantified (occludin, claudin 1, claudin 2). The mean pixel intensity was used to quantify each region that overlapped with occludin labeling.

### Western blotting

Fresh human skin tissues were cut into very small pieces. The appropriate amount of cytoplasmic protein extract was then mixed in. The tissue was homogenized in RIPA buffer (Servicebio). The protein concentrations were determined with a BCA protein concentration measurement kit. The skin samples were then separated by SDS–PAGE and electrotransferred to polyvinylidene fluoride (PVDF; 0.45 μm) membrane (Roche, Basel, Switzerland) at 300 mA for 30 min. The membrane was then blocked in a TBS buffer containing 3% BSA for 30 min at 37°C. Then, the membrane was incubated with primary antibodies at 4°C overnight and HRP-conjugated secondary antibodies for 30 min. The results from the two groups were compared within the software ImageJ.

### RNA preparation and quantitative real-time PCR

Real-time PCR was used to investigate the total amount of RNA isolated from the normal and blistered skin samples. The isolated RNA was quantified, reverse transcribed, and analyzed by qRT–PCR (Stepone plus, ABI, United States). The PCR primer sequences for occludin were 5′-TTC​CTA​TAA​ATC​CAC​GCC​GG-3′ (forward) and 5′-TGT​CTC​AAA​GTT​ACC​ACC​GCT​G-3′ (reverse); those for claudin 1 were 5′-CTG​TGG​ATG​TCC​TGC​GTG​TC-3′ (forward) and 5′-ACT​GGG​GTC​ATA​GGG​TCA​TAG​AAT (reverse); those for claudin 2 were 5′- TTG​GGC​TTG​GTA​GGC​ATC​GT-3′ (forward) and 5′- CAG​GAA​TCC​CGA​GCC​AAA​GA (reverse); and those for β-actin were 5′- CAC​CCA​GCA​CAA​TGA​AGA​TCA​AGA​T-3′ (forward) and 5′- CCA​GTT​TTT​AAA​TCC​TGA​GTC​AAG​C-3′ (reverse). The cycling conditions were set as follows: 25°C for 5 min, 42°C for 30 min, and 45 cycles of 85°C for 5 s. The mRNA levels were normalized to the corresponding β-actin levels in human skin.

### Statistical analysis

Continuous data are presented as the means with standard deviation. Mann–Whitney U tests were conducted for comparisons between the two independent groups, and Kruskal–Wallis H tests were used to conduct comparisons among three independent groups. Homogeneity of variance for continuous variables was evaluated using the Levene test for equality of variances. For all analyses in this research, significance was set at the *p* < 0.05 level. All analyses were conducted using SPSS version 22.0 (IBM Corp, Armonk, NY).

## Results

### Human blistered skin demonstrated a decrease in epidermal differentiation markers

**FIGURE 3:**
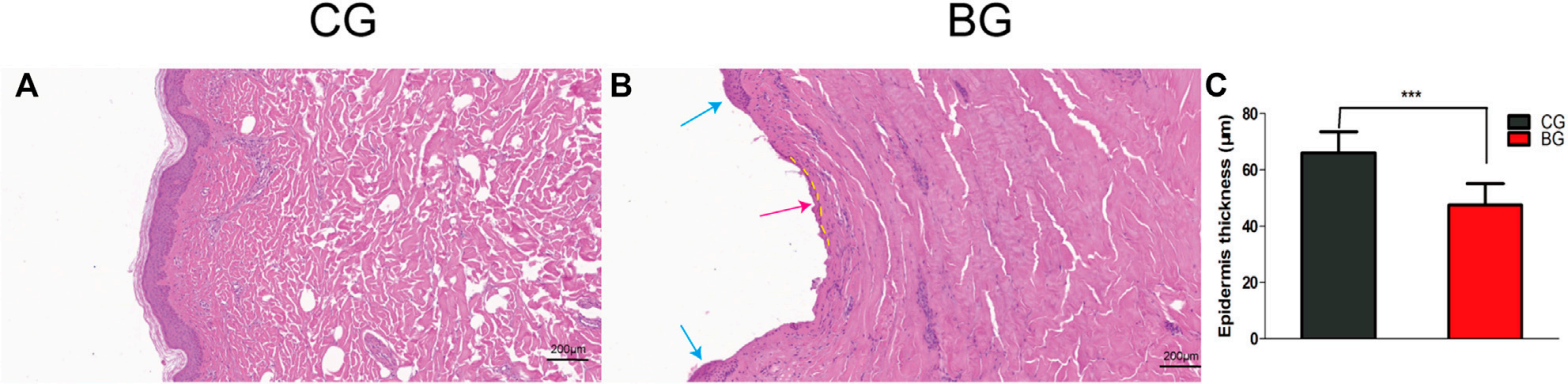
**(A)** Normal skin was stained with HE, and the cornified layer in the epidermal layer was intact. **(B)** Blistered skin was stained with HE and observed by light microscopy. The epidermal layer was disrupted (blue arrow) and separated from the dermis, with a small portion of the stratum basale retained (dotted yellow line), which explained the quick recovery of the blistered skin (red arrow). **(C)** Comparison of skin thicknesses in the two groups. CG: control group; BG: blister group.

Compared with the normal corneum stratum in the normal skin, the cornified layer was completely disrupted and disappeared in blistered skin, as observed by the histology of HE-stained skin. The epidermis was separated from the dermis, and a portion of the stratum basale was retained (Figure 3). The thickness of the epidermis was significantly decreased in the BG compared with that in the CG (*p* < 0.001).

**FIGURE 4:**
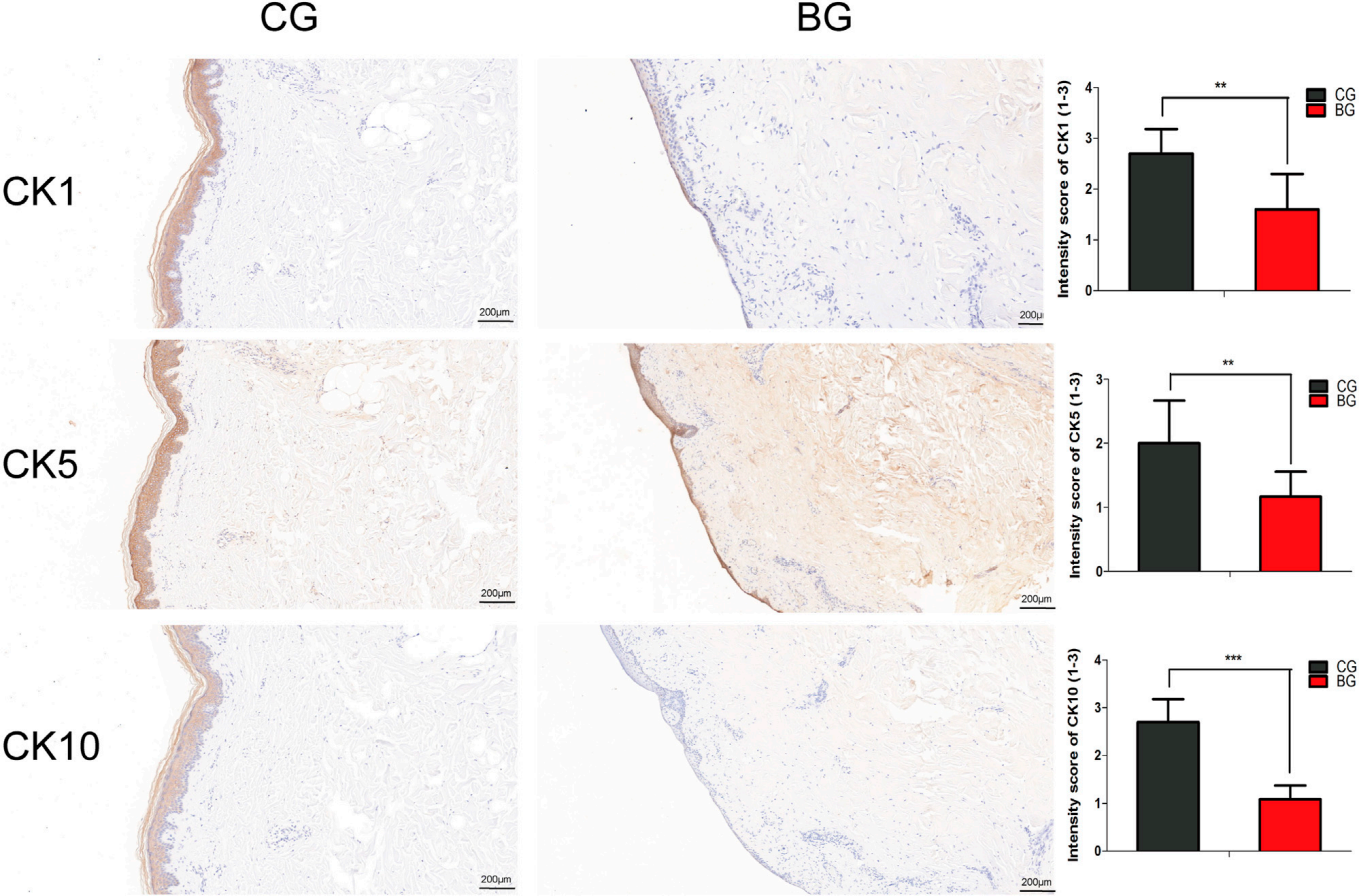
Immunohistochemical images of the two groups with antibodies against cytokeratins K1, K5, and K10. The epidermal layer was disrupted and separated from the dermis, while a small portion of the stratum basale was retained in the BG. The cytokeratins were distributed nonhomogeneously in the residual middle layer of the epidermis. The intensity scores for K1, K5, and K10 in the two groups were compared, and the expressions of K1, K5, and K10 were significantly decreased in the BG. K1: cytokeratin 1, K5: cytokeratin 5, K10: cytokeratin 10. (Bars = 200 μm, *: *p* < 0.05, **: *p* < 0.01, and ***: *p* < 0.001).

**FIGURE 5:**
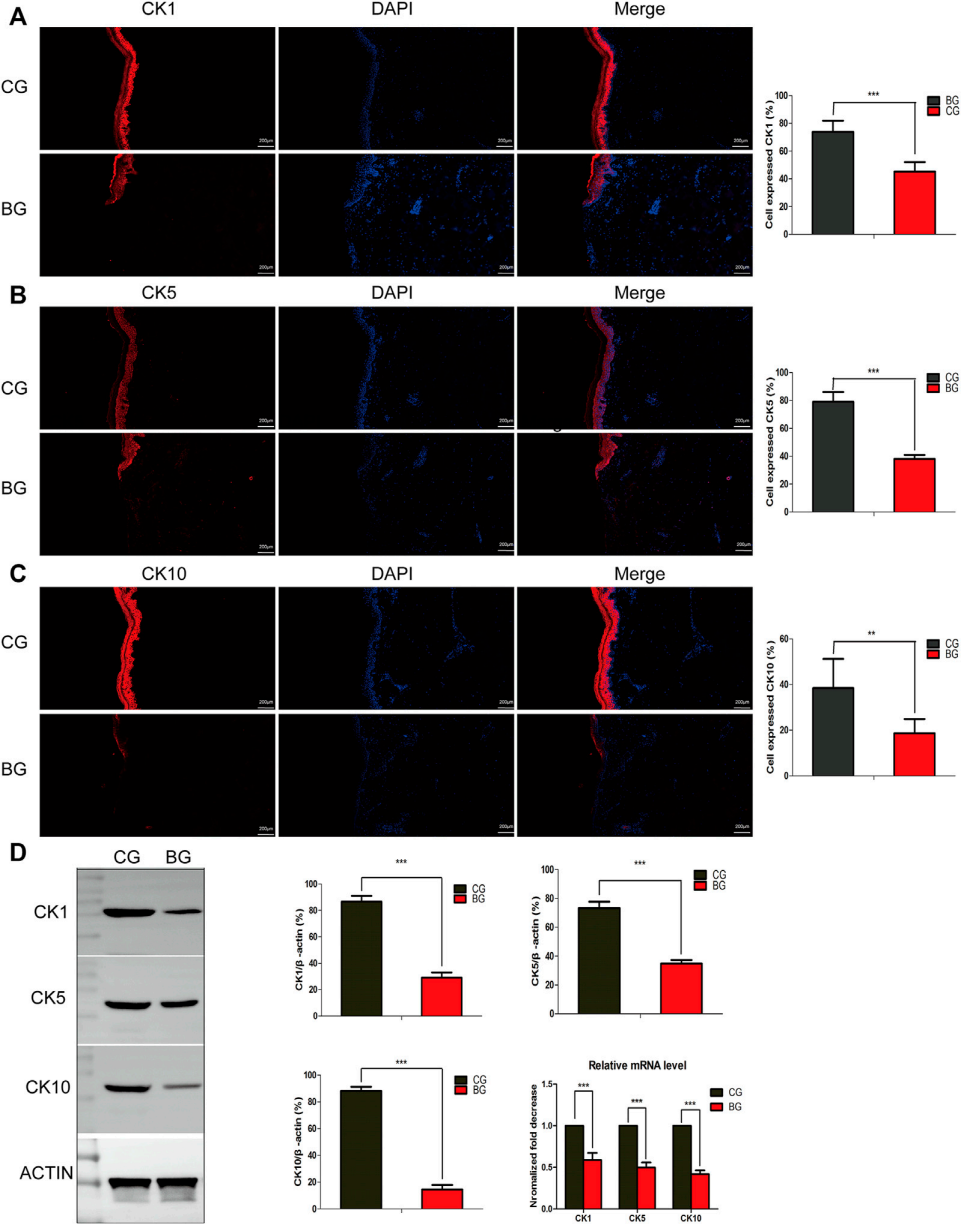
**(A–C)** Immunofluorescence analysis of normal and blistered skin. Images of immunofluorescence staining of the cytokeratins K1, K5, and K10 in the two groups. In accordance with the immunohistochemical results, the number of positively expressing cells was compared for K1, K5, and K10, and the positive expression rate was significantly decreased in the BG. **(D)** Representative Western blots of cytokeratins K1, K5, and K10 in control and blistered human skin. It was observed that the expression of the three cytokeratins was significantly decreased in the BG group. *: *p* < 0.05, **: *p* < 0.01, and ***: *p* < 0.001).

To further analyze the changes after blister observation, normal and blistered skin were analyzed with immunohistochemistry against cytokeratin 1 (K1), 5 (K5), and 10 (K10). It was demonstrated that in blistered skin, K1, K5, and K10 were distributed nonhomogeneously in the residual middle layer of the epidermis, namely, the spinous layer and granular layer, while control tissues showed that the expression was distributed uniformly within the intact middle layer. Furthermore, there was no significant difference in localization, but the intensity decreased in blistered skin (Figure 4). IF analysis (Figures 5A–C), Western blotting, and PCR (Figure 5D) further confirmed these results. The expressions of K1, K5, and K10 were decreased in blistered skin compared with normal skin.

### Phosphatidylinositol-3-kinase/protein kinase B expression was upregulated, and epidermal expression of tight junctions was decreased in blistered skin

**FIGURE 6:**
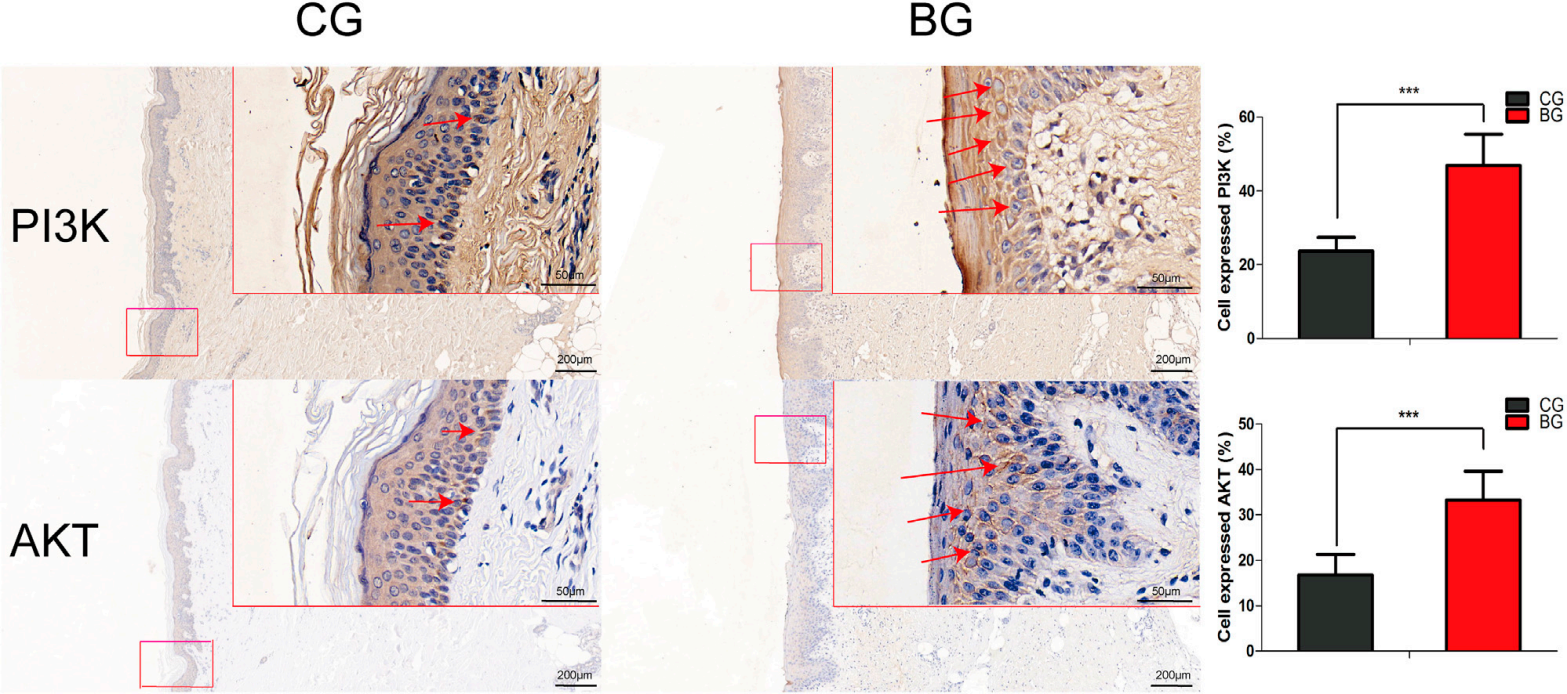
Immunohistochemical images of PI3K and AKT in normal and blistered skin. The number of cells expressing PI3K or AKT in the two groups was compared, and the expression of PI3K or AKT increased significantly in blister skin compared with normal skin. (Bars = 200 and 50 μm, *: *p* < 0.05, **: *p* < 0.01, and ***: *p* < 0.001).

**FIGURE 7:**
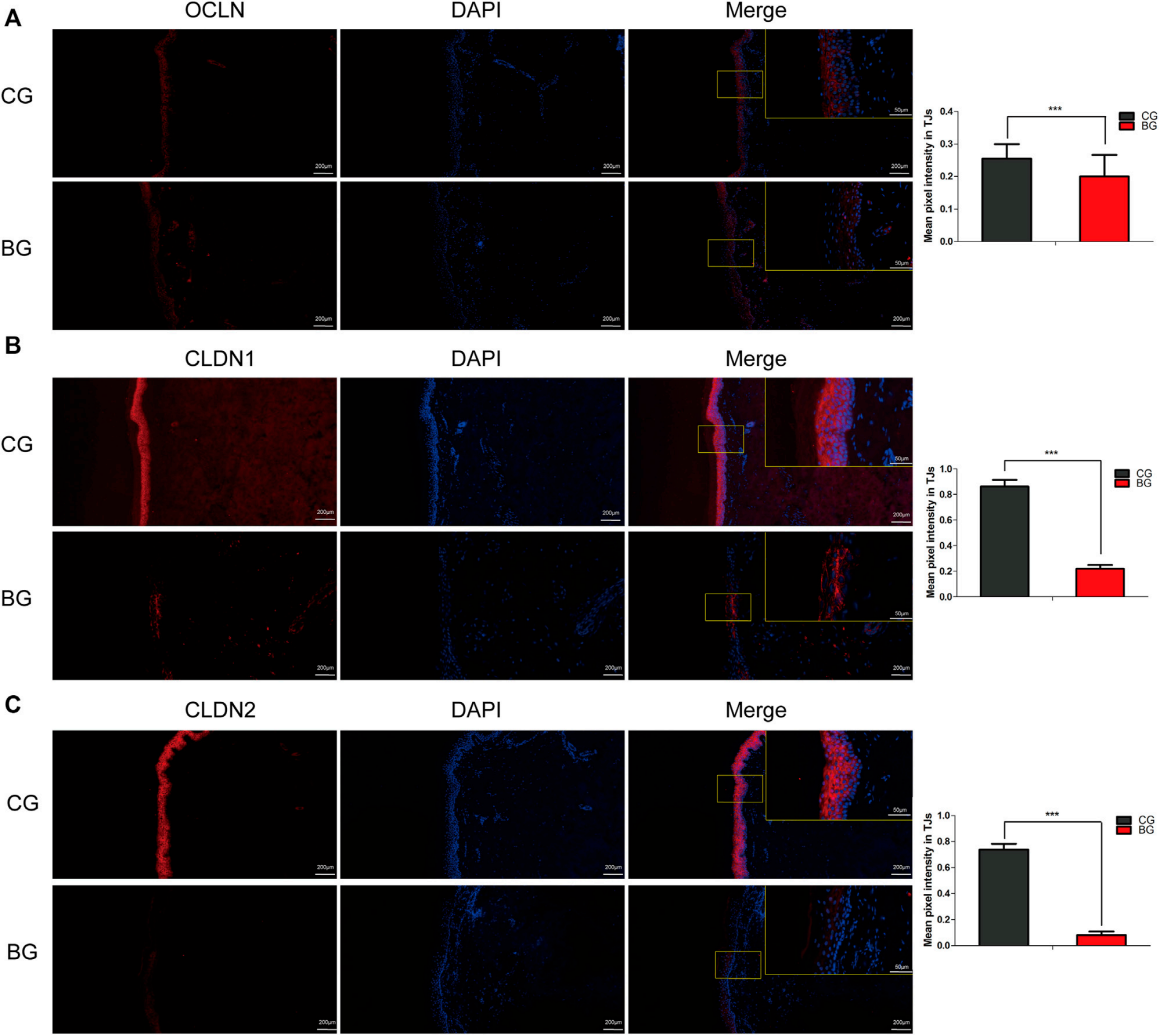
Immunofluorescence images of occludin, claudin 1, and claudin 2 in normal and blistered skin. The nuclei were stained with DAPI (blue), and red fluorescence indicates the lineage markers. The OCLN **(A)**, CLDN1 **(B)**, and CLDN2 **(C)** levels in each group were compared, and it was illustrated that the expression of these TJ markers decreased significantly in blister skin compared with normal skin. OCLN: occludin, CLDN1: claudin 1, CLDN 2: claudin 2. (Bars = 200 and 50 μm, *: *p* < 0.05, **: *p* < 0.01, and ***: *p* < 0.001).

Immunohistochemistry with antibodies against PI3K or AKT was conducted, and the expressions of PI3K and AKT increased significantly in blistered skin compared with normal skin (Figure 6). To further analyze the effects of blisters on the epidermis, the treated tissues were subjected to IF staining with antibodies against target TJ proteins, including claudin 1, claudin 2, and occludin. As a type of TJ protein, the expression of occludin was decreased significantly in blistered skin but normally distributed in normal skin. The expression of claudin 1 and 2 also decreased significantly in blistered skin compared with the control group (Figure 7).

### Blistered skin exhibited downregulated occludin and claudin 1 and 2 protein expressions and increased phosphatidylinositol-3-kinase/protein kinase B pathway proteins

**FIGURE 8:**
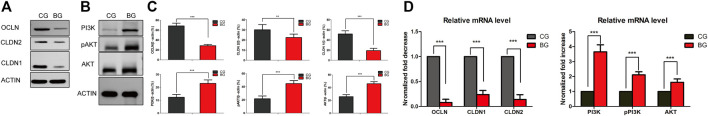
**(A)** Representative Western blots of the proteins OCLN, CLDN1, and CLDN2 in control and blistered human skin. **(B)** Representative Western blots of the proteins PI3K, AKT, and P-AKT in control and blistered human skin. **(C)** Expressions of the OCLN, CLDN1, and CLDN2 proteins were significantly decreased and those of PI3K, AKT, and pAKT were significantly increased in blistered skin compared with normal skin. OCLN: occludin, CLDN1: claudin 1, CLDN 2: claudin 2. (*: *p* < 0.05, **: *p* < 0.01, and ***: *p* < 0.001). **(D)** mRNA expression levels of occludin and claudins 1 and 2 were measured by qRT–PCR, and it was found that occludin, claudin 1, and claudin 2 expressions were decreased in blistered skin. In contrast, the mRNA expression levels of PI3K, p-PI3K, and AKT were significantly increased in blistered skin. The results are expressed as the mean ± SEM. ***: *p* < 0.001.

Occludin and claudins 1 and 2 are signaling molecules in the skin barrier. Western blot analysis revealed that the protein expressions of occludin and claudins 1 and 2 in the blister group were markedly decreased compared to those in the control group. Furthermore, Western blot analysis of PI3K, pPI3K, and AKT was performed and the expression of PI3K and AKT was found to have increased compared with that in the control group. These results further suggested that the blisters observed in the skin after tibial plateau fracture could contribute to increased fluid accumulation in human skin (Figures 8A–C).

### Blistered skin exhibited downregulated occludin and claudins 1 and 2 mRNA expressions

To investigate the effect of the blisters on epidermal or dermal differentiation, the mRNA levels of occludin and claudins 1 and 2, which are proteins involved in the formation of the epidermal barrier, were evaluated using qPCR. The expressions of occludin and claudins 1 and 2 mRNA were found to be significantly decreased in blistered skin. The mRNA expression levels of PI3K, p-PI3K, and AKT were significantly increased in blistered skin (Figure 8D).

## Discussion

Patients with severe tibial plateau fractures (Schatzker V and VI) were enrolled in this study due to the relatively severe fracture pattern and the fact that more blisters are observed in these patients than in patients with other tibial plateau fractures or fractures in other areas. Undoubtedly, blisters have always been a recognized entity observed on human skin, but blisters observed above tibial plateau fractures have continually been ignored in orthopedic studies. Fracture blisters are known to be associated with increased infection rates and wound breakdown, and the current research focused on the pathological characteristics of blistered skin has attempted to illustrate skin changes and determine the potential mechanism of fracture blister fluid formation. The expression of TJs was significantly decreased in blistered skin, and the paracellular pathway was opened, which contributed to the formation of blister fluid. This study opens a new door for understanding and explaining fracture blisters, which have been associated with reduced compartment pressure in the clinic.

Epithelia are sheets of cells that line body cavities and external surfaces in multicellular organisms. One key function of the epidermis is to act as a physical and chemical barrier that allows the selective transportation of solutes and water between compartments (Günzel and Yu, 2013). However, the definite mechanism of fracture blister fluid formation is still not clear, and many phenomena cannot be reasonably explained. First, it was found that manual intradermal injection of fluid under maximal pressure with a syringe did not result in blister formation (Stoughton, 1957). Furthermore, equal high pressures have also been observed in the corium in diseased states. Therefore, it seems very unlikely that the pressure itself can directly cause blister formation. Second, the blisters resulting from friction do not cause the direct separation of epidermal cells from the corium, which is actually secondary to the cell damage incurred by friction (Stoughton, 1957). Third, Bork (1978)reported that negative interstitial fluid pressure and the colloid osmotic pressure of the interstitial fluid were the main factors affecting blister fluid formation. However, the amount of fluid in the blister was potentially large, and the fast accumulation of blister fluid cannot be reasonably explained by only intracellular filtration pressure. Therefore, an understanding of the fluid transport mechanism in the skin is essential and beneficial to revealing the mechanism of fluid accumulation and decreased compartment pressure when blisters are observed in the skin above severe tibial plateau fractures.

Many factors can affect the permeability of the skin; for example, epidermal integrity can be disrupted by disulfide bond cleavage agents such as sodium lauryl sulfate and hydrogen bond breaking agents. It has also been reported that thermal injury can release enzymatic factors that cause the breakdown of intercellular bridges and result in epidermal cells separating from each other. Furthermore, the blood–brain barrier consists of TJs between endothelial cells that inhibit the transcellular or paracellular passage of molecules across it, and it was reported that circulating TJs were increased when the integrity of the blood–brain barrier was disrupted (Kniesel and Wolburg, 2000; Jiao et al., 2015). Similarly, epidermal cells are also attached to each other at their lateral membranes through a complex of intercellular junctions, such as TJs or zona occludens, and TJs are considered the major determinant of paracellular permeability (Stoughton, 1957; Ahmad et al., 2011). Researchers studying epithelial transport and TJs, which have always been considered to be impermeable structures, noted that they are actually variably permeable to ions and solutes (Van Itallie and Anderson, 2014). Consistent with the aforementioned research, it was found that the blistered skin in tibial plateau fractures had significantly decreased expression of TJ proteins such as claudins 1 and 2 and occludin. Along with the decreased expression of TJs, the AKT signaling pathway is considered to play an important regulatory role in the formation and maintenance of TJs. The results here illustrated that the expression of PI3K and AKT was increased in blistered skin compared with that in the control group, which indicates the potential role of PI3K in regulating the expression of TJs. Immunohistochemical images also showed that the staining intensity of cytokeratins 1, 5, and 10 decreased or even disappeared, reflecting that the epidermal integrity was disrupted in blistered skin. It was also found that some residual epidermis of the stratum basale was retained with the decreased TJ expression. Therefore, after understanding the importance of TJs in the paracellular pathway of skin, it was concluded from our experimental results that decreased TJ expression in blistered skin was beneficial to the paracellular transportation of ions and solutes.

Due to the structural changes in blistered skin, fluid can migrate from the interstitial space into the blister space through the disrupted TJs. To achieve migration, physical forces that act on the interstitial fluid in the interstitial space must be present. What we hypothesized was that the high compartment pressure in a tibial plateau fracture, which is due to abnormal physical forces, might be conducted through the fascia tissue, muscles, and hypodermis to the interstitial space under the skin. Then, the fluid is pushed into the blisters, although transcellular or paracellular pathways adjusted after tight conjunction expressions were decreased during the process of fracture blister fluid formation. As the volume of fluid in the blister increases, the compartment pressure decreases. Furthermore, blood blisters are always observed in the clinic and can actually be identified as a marker of the disruption of TJs. Electron-dense molecules such as hemoglobin are not permeable through the paracellular pathway under normal conditions. Studies have also shown that hemoglobin freely diffuses through the intercellular space but stops at the level of the TJ (Farquhar and Palade, 1963; Martinez-Palomo et al., 1971; Whittembury and Rawlins, 1971). Therefore, TJ disruption in blistered skin can be identified as an essential mechanism to accelerate fluid accumulation in blisters and indirectly decrease compartment pressure.

There are limitations to this research. One is that fluid migration was not monitored in real-time, and in our following research, tracer agents such as ruthenium red will be added to illustrate fluid transportation. However, experiments in humans are not easily conducted, so related animal experiments will be used to test the validity of our hypothesis. Second, related transcellular pathways were not explored here, and piezo or transient receptor potential vanilloids will be detected in our subsequent research in fascia and skin tissue samples. Crushing injury or vascular injury was also not considered in this study. To obtain a more comprehensive understanding of ACS, more patients and other injury mechanisms will be compared in our follow-up research.

## Conclusion

In conclusion, the epidermal layer was disrupted and separated from the dermis with a portion of stratum basale residue, and the expression of TJ proteins such as claudins and occludin decreased significantly in the blistered skin, thus resulting in reduced barrier function. The changes in TJs after blister observation may be a potential channel for explaining blister fluid formation, which indirectly explains the decreased compartment pressure under blistered skin after severe tibial plateau fracture. The paracellular pathways regulated by TJs are regulated under physiological conditions and affected under pathological fractured states through the PI3K/AKT pathways.

## Data Availability

The original contributions presented in the study are included in the article/supplementary material; further inquiries can be directed to the corresponding authors.
